# Identification and characterisation of the novel endogenous promoter *HASP1* and its signal peptide from *Phaeodactylum tricornutum*

**DOI:** 10.1038/s41598-019-45786-9

**Published:** 2019-07-09

**Authors:** Erdenedolgor Erdene-Ochir, Bok-Kyu Shin, Byeori Kwon, Choonkyun Jung, Cheol-Ho Pan

**Affiliations:** 1 Natural Product Informatics Research Center, KIST Gangneung Institute of Natural Products, Gangneung, 25451 Republic of Korea; 2 Division of Bio-Medical Science and Technology, KIST School, Korea University of Science and Technology, Seoul, 02792 Republic of Korea; 3 Algaeprona Inc, Gangneung, 25451 Republic of Korea; 4 Graduate School of International Agricultural Technology and Crop Biotechnology Institute/GreenBio Science and Technology, Seoul National University, Pyeongchang, 25354 Republic of Korea

**Keywords:** Marine biology, Reporter genes, Reporter genes, Marine biology, Expression systems

## Abstract

Although diatoms have been extensively studied as bioreactors, only a limited number of efficient gene promoters are available. Therefore, the development of new endogenous promoters is important for the heterologous production of a variety of recombinant proteins. Herein, we identified the most abundant secreted protein in *Phaeodactylum tricornutum*, designated ‘highly abundant secreted protein 1’ (HASP1), and characterised the activities of its promoter and signal peptide using green fluorescent protein (GFP) as a reporter. The *HASP1* promoter strongly drove GFP expression during all growth phases of *P*. *tricornutum* in culture, in contrast to the commonly used *fcpA* promoter, which is less active during the stationary phase. The HASP1 signal peptide was also sufficient for facilitating efficient secretion of GFP by *P*. *tricornutum*. Our findings suggest that both the promoter and the signal peptide of HASP1 can be utilized as novel tools for the overexpression and secretion of recombinant proteins in *P*. *tricornutum*.

## Introduction

Diatoms are unicellular, eukaryotic phytoplankton that inhabit marine and freshwater environments and are responsible for about 20% of the primary photosynthetic productivity on earth^1^. Compared with other microalgae, diatoms have a higher carbon fixation ability^2^. *Phaeodactylum tricornutum* is a pleiomorphic coastal marine diatom that utilizes silicic acid in a facultative manner^3^. The complete genome of *P*. *tricornutum* is approximately 27.6 Mb in size and consists of 33 chromosomes containing 12,177 predicted protein-coding genes^4^. A large number of expressed sequence tags (ESTs) have been identified in *P*. *tricornutum* cells grown under various different conditions, including different sources of nutrients, morphotypes, lighting sources, and abiotic stresses^5,6^. Furthermore, tools for genetic manipulation of *P*. *tricornutum*, such as gene overexpression, gene silencing, gene editing, and plasmid delivery methods, are well established^7–11^.

Diatoms are being actively studied for diverse biotechnological applications and can currently be used as bioreactors for the production of biopharmaceuticals and secondary metabolites. For example, *P*. *tricornutum* has been genetically engineered to produce pharmaceutically important proteins such as human IgG antibodies^12^. In this regard, the N-glycosylation pathways in *P*. *tricornutum* can be remodelled to have a human-compatible glycosylation profile^13^. The development of an optimised expression system using a suitable promoter is an essential prerequisite for heterologous gene expression. A strong promoter that drives high yields of recombinant protein is not only crucial for developing a cost-effective expression system but is also indispensable for metabolic engineering through gene regulation. The endogenous promoter from the gene encoding fucoxanthin chlorophyll a/c binding protein (FCP) has been commonly used for both genetic and metabolic engineering of *P*. *tricornutum* for a variety of different applications^14–18^. However, the *fcpA* promoter is light-inducible and is not always able to drive constitutive expression of downstream transgenes throughout all growth phases of the culture^19–21^. The nitrate reductase gene promoter has also been widely used as a strong endogenous promoter for many purposes, such as the heterologous expression of human antibodies^12^. However, the disadvantage of the nitrate reductase promoter is that it requires an exogenous nitrogen source to induce downstream gene expression. Recently, other endogenous promoters such as *V-ATPase C* and *Pt211* have been identified as strong promoters^22,23^. On the other hand, heterologous promoters, such as the *CIP1* promoter of the putative replication-associated protein gene from the *Chaetoceros lorenzianus*-infecting DNA virus, are less active than the diatom endogenous gene promoter^19,24^.

The secretion of recombinant proteins into the culture medium is a powerful and cost-efficient platform for producing pharmaceutically important proteins because the steps needed to purify secreted recombinant proteins are less complicated than the steps needed for the purification of intracellular proteins. Endogenous signal peptides from the ARS1 and gametolysin proteins have been used to promote secretion of recombinant proteins into the culture medium in *Chlamydomonas reinhardtii*^25,26^. A human IgG antibody without an endoplasmic reticulum retention signal has also been shown to be efficiently secreted into the culture medium of *P*. *tricornutum*^27^. Despite their great advantages, protein secretion pathways have been rarely investigated in *P*. *tricornutum*.

In this study, we performed a secreted proteome profile study of *P*. *tricornutum* using liquid chromatography tandem mass spectrometry (LC-MS/MS) to examine the secreted proteins present in the culture medium. We first identified several hundred proteins that are secreted at high levels into the extracellular space in the stationary phase and designated these as HASPs (highly abundant secreted proteins) for further analysis. Among these, we found that the HASP1 protein was the most abundant protein in the culture medium. In order to develop a novel expression system for producing secreted recombinant proteins in *P*. *tricornutum*, we characterised promoter and its signal peptide using green fluorescent protein (GFP) as a reporter. The *HASP1* promoter strongly drove GFP expression, especially in the stationary phase of *P*. *tricornutum* culture, to a much higher level than that of the *fcpA* promoter. The signal peptide of the HASP1 protein was sufficient to result in GFP secretion into the culture medium of *P*. *tricornutum*. Taken together, our findings provide evidence that the endogenous *HASP1* promoter is a novel strong promoter that can be used as a suitable tool for overexpressing recombinant proteins in *P*. *tricornutum*.

## Results and Discussion

### Identification of highly abundant secreted proteins in stationary phase cultures of *P*. *tricornutum*

**Figure 1:**
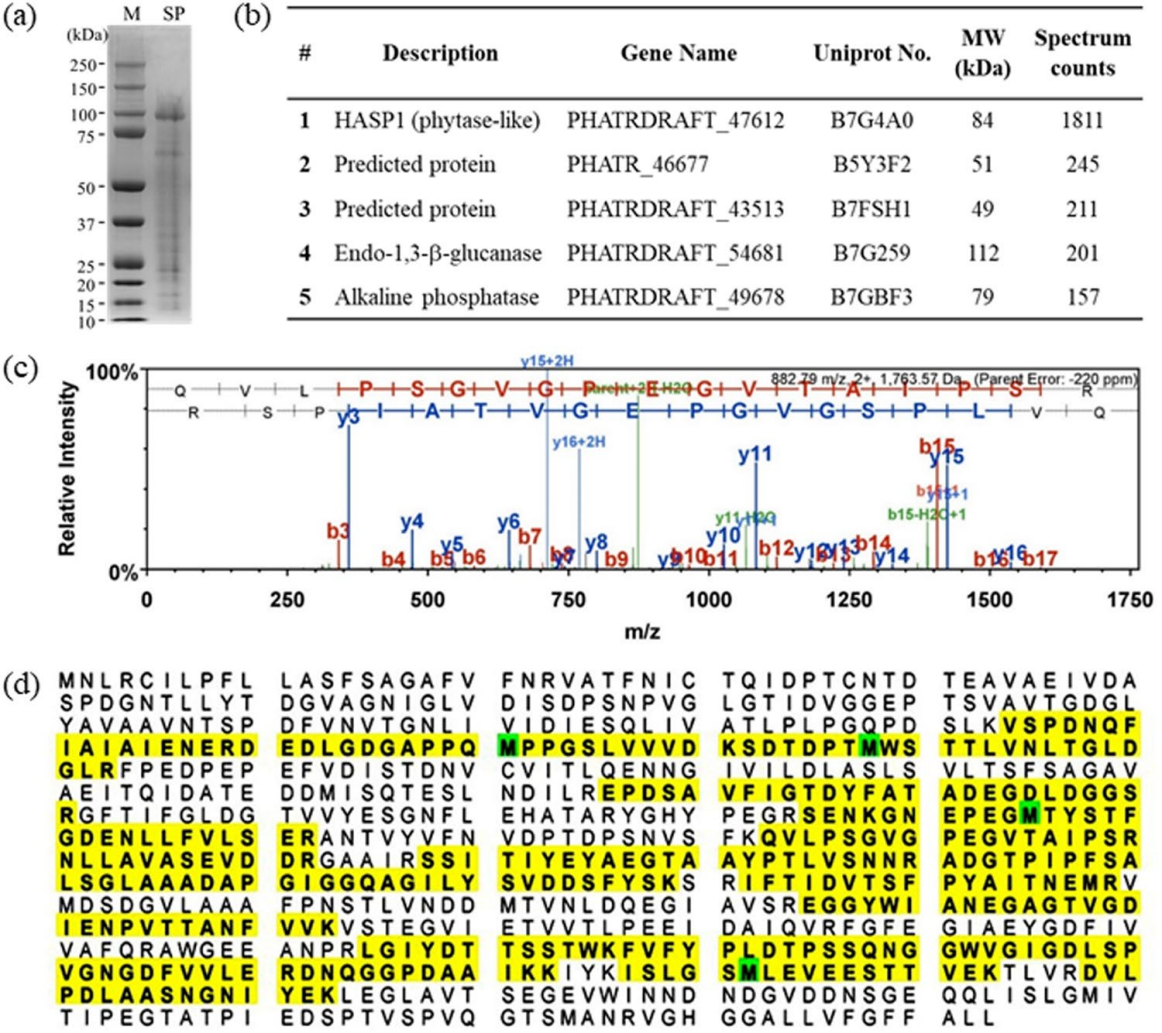
Secreted proteome profiling of *P*. *tricornutum* using LC-MS/MS. (**a**) Abundantly secreted proteins were separated by SDS-PAGE and visualised by staining with colloidal Coomassie blue. M, molecular mass marker; SP, secreted protein. (**b**) List of the five highest abundant proteins in the culture supernatants identified by LC-MS/MS. MW, molecular weight. (**c**) Mass spectra of the HASP1 protein. (**d**) Sequence coverage of the HASP1 protein by LC-MS/MS analysis. Yellow highlighted sequences show the peptide sequences identified by LC-MS/MS analysis (44% coverage, 345/793 amino acids). Green highlighted M, oxidation.

**Figure 2:**
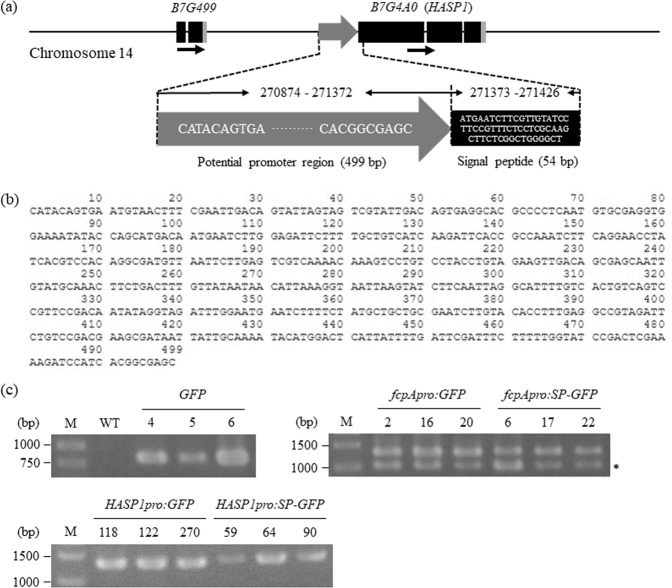
Isolation of the potential promoter and putative signal peptide of the *HASP1* gene and selection of transformants by PCR analysis with genomic DNA. (**a**) Schematic representation of the *HASP1* gene location on chromosome 14. The grey arrows indicate the potential promoter of the *HASP1* gene. Black arrows indicate the gene orientation. (**b**) Nucleotide sequence of the potential promoter region of the *HASP1* gene. (**c**) PCR amplification of transgenes from the genomic DNA of transformants. The numbers indicate independent transgenic lines for each construct. Full-length agarose gels are presented in Supplementary Fig. S4. Asterisk shows the nonspecific PCR products. M, molecular size marker.

The abundantly secreted proteins in the stationary phase culture medium of *P*. *tricornutum* were resolved by SDS-PAGE and visualised by staining with colloidal Coomassie blue (Fig. 1a). LC-MS/MS analysis of the most abundant secreted proteins identified a total of 468 proteins. The five highest secreted proteins, whose relative abundance in the culture medium was compared based on their spectral counts in LC-MS/MS analysis, are shown in Fig. 1b. Among these, we found that the most abundantly secreted protein was PHATRDRAFT_47612 (NCBI accession number: XM_002181840.1), identified through database searching with a 44% sequence coverage; we have designated this protein as HASP1 (highly abundant secreted protein 1; consists of 793 amino acids; Fig. 1c,d). The HASP1 protein contains a phytase-like domain in the C-terminal region and therefore belongs to the phytase superfamily. The fourth most abundant protein (PHATRDRAFT_54681) was annotated via homology as the enzyme endo-1,3-beta-glucanase which is involved in carbohydrate metabolism^28^. In addition, the fifth most abundant protein (PHATRDRAFT_49678) encodes an alkaline phosphatase, whose activity has been experimentally proven^29^. The protein levels of HASP1 were drastically higher than those of any of the other protein in the culture medium. Therefore, we selected the HASP1 protein for further analysis and isolated its potential promoter region and putative signal peptide (Fig. 2a,b) to test their effects on the production and secretion of recombinant proteins in *P*. *tricornutum*. The *fcpA* promoter, a widely used promoter in *P*. *tricornutum*, was used as a control^30^.

### Isolation of the potential promoter and the putative signal peptide of the *HASP1* gene

The *HASP1* gene is located on chromosome 14 of the *P*. *tricornutum* genome^31^. The intergenic region between *HASP1* and the upstream Phatrdraft_52260 gene was 3,534 bp long, extending from after the stop codon of Phatrdraft_52260 to before the start codon of *HASP1* (Fig. 2a). The *P*. *tricornutum* genome has a relatively higher gene density than other higher eukaryotic genomes^32^. Approximately, 500-bp upstream regions of endogenous genes are sufficient for full promoter activity by strongly driving transgene expression^7,12,15,19,22,33^. Therefore, we considered the 499-bp upstream region before the start codon of the *HASP1* gene as the potential promoter. The *HASP1* potential promoter contains a 5′-untranslated region, an initiator-like sequence, and potential *cis*-acting regulatory elements that are recognised by transcription factors such as Myb and bZIP (Supplementary Fig. S1)^24,29,32,34–39^. Using SignalP software, the 18-amino acid long signal peptide (SP) responsible for HASP1 secretion was found in the N-terminus^33^ (Fig. 2b). The nucleotide sequences of the 499-bp long *HASP1* potential promoter and the putative signal peptide were obtained using the Biomart tool in EnsemblProtists^40^. The potential promoter fragment of *HASP1* and the *fcpA* promoter were amplified by PCR and then cloned into a modified pPha-T1 vector to drive GFP expression (*HASP1pro:GFP* and *fcpApro:GFP*). The putative signal peptide of the HASP1 protein was fused in frame to the N-terminus of GFP to allow for secretion of the GFP protein into the extracellular space (*HASP1pro:SP-GFP* and *fcpApro:SP-GFP*). The GFP levels in the supernatant of *P*. *tricornutum* cultures, either with or without the putative signal peptide, were compared to assess the efficiency of GFP secretion into the culture medium. It should be noted that in order to enhance GFP expression, a minimal Kozak sequence (ACC) was placed directly before the putative signal peptide derived from the HASP1 protein. In contrast, this Kozak sequence was not inserted into the vectors lacking the putative HASP1 signal peptide. A promoter-less vector harbouring only the *GFP* gene was used as a negative control (Supplementary Fig. S2).

### Transformation of *P*. *tricornutum* and selection of transformants

*P*. *tricornutum* cells grown to the stationary phase were transformed with the five different constructs (*fcpApro:SP-GFP*, *HASP1pro:SP-GFP*, *fcpApro:GFP*, *HASP1pro:GFP*, and *promoter-less GFP*) by particle bombardment. Putative transformants were selected on f/2 agar medium containing zeocin for at least 3 to 4 weeks. Following this, the zeocin-resistant colonies were transferred to the same medium for further growth, and their zeocin resistance was reassessed and confirmed. The presence of the transgene in the genome of the putative transformants was confirmed by PCR using *P*. *tricornutum* genomic DNA as the template. More than 400 colonies were grown and selected on zeocin-containing medium; subsequently, at least 70% of the zeocin-resistant colonies were found to have the corresponding transgene in their genomes. We selected three independent transgenic lines from each construct for further analysis; PCR analysis results for each transgene are shown in Fig. 2c (Uncropped agarose gel images used to generate Fig. 2c are shown in Supplementary Fig. S4).

### Quantification of the *HASP1* promoter activity driving *GFP* expression

**Figure 3:**
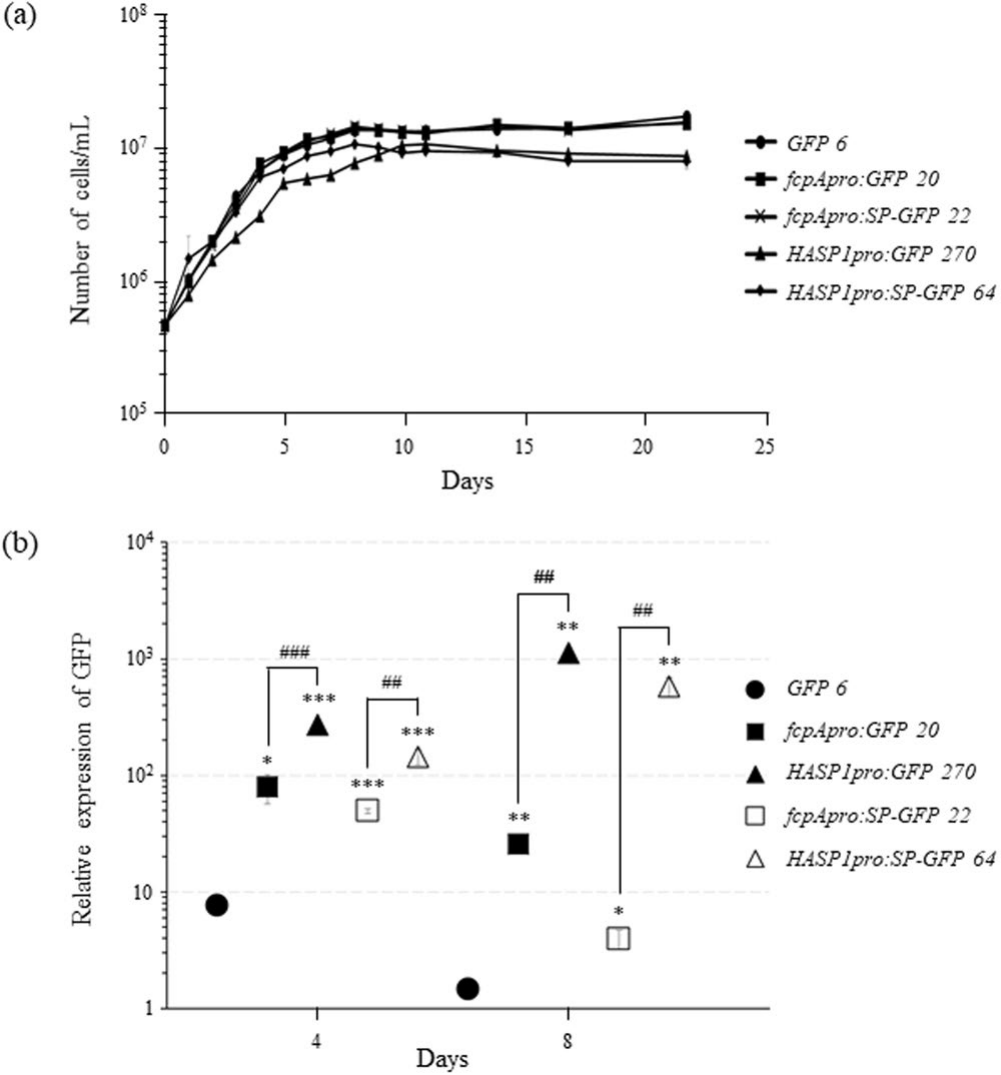
Growth curves of transgenic *P*. *tricornutum* and levels of GFP transcript in the selected transgenic lines. (**a**) Cell growth curves of *P*. *tricornutum* cultures. The selected transgenic lines were grown for 22 days. (**b**) Relative levels of GFP mRNA in the selected transgenic lines. GFP expression levels were normalised to *TBP* (TATA-box binding protein) expression. Data are expressed as the mean ± SD of three replicates. The asterisk (*) and hash (#) indicate statistically significant differences vs. the transformant groups of the negative (promoter-less *GFP*) and positive controls, respectively. ns, not significant (*p* > 0.05); *^/#^*p* < 0.05, **^/##^*p* < 0.01, ***^/###^*p* < 0.001 (ANOVA).

Transgenic *P*. *tricornutum* cells were seeded at a density of 5 × 10^5^ cells/mL and grown for 22 days with a periodic assessment of cell number to generate growth curves (Fig. 3a). From the *P*. *tricornutum* culture growth curves of all transgenic lines, it took approximately 4 and 8 days to reach the log and stationary phases, respectively. For all transgenic lines, the cell densities exceeded 1 × 10^7^ cells/mL at the stationary phase. It should be noted that the transgenic lines harbouring the *HASP1* promoter showed slightly slower growth than that of the other transgenic lines. Nonetheless, the data indicated that in all cases, transgene presence did not have any harmful effects on the growth rate of *P*. *tricornutum* cells. We then extracted total RNA from each transgenic cell line at both the log (day 4) and stationary phases (day 8) to compare the activity of the *HASP1* promoter with that of the *fcpA* promoter by measuring the levels of *GFP*-containing transcripts by real-time RT-PCR. The levels of *GFP*-containing transcripts in the transgenic line expressing *HASP1pro:GFP* were 3 and 44-fold higher than those expressing *fcpApro:GFP* on day 4 and 8, respectively. When compared with the promoter-less *GFP* control, the levels of *GFP* expression driven by the *HASP1* promoter increased by 35 and 764-fold on day 4 and 8, respectively (Fig. 3b). It is well known that the *fcpA* promoter is primarily active during the log phase^19^. Notably, the *HASP1* promoter activity was higher than that of the *fcpA* promoter during the same phase. In addition, with respect to the level of *GFP* transcripts, *HASP1* promoter activity was maintained at an even higher level in the stationary phase than in the log phase. These results are consistent with expression levels of endogenous *fcpA* and *HASP1* genes. In 8-day grown wild-type *P*. *tricornutum* cells, the level of endogenous *HASP1* mRNA was approximately 40-fold higher than that of endogenous *fcpA* mRNA. (Supplementary Fig. S3).

We have therefore demonstrated that—compared with a previously reported promoter—the *HASP1* promoter is more active in *P*. *tricornutum*^7,11,19,24,41–45^. Moreover, the transcriptional level of *SP-GFP* in the *HASP1pro:SP-GFP* line was 3 and 147-fold higher than that of the *fcpApro:SP-GFP* line on day 4 and 8, respectively. *SP-GFP* transcript levels driven by the *HASP1* promoter were 19 and 395-fold higher than those of the promoter-less *GFP* control on day 4 and 8, respectively (Fig. 3b). Although the mRNA levels of *SP-GFP* driven by the *HASP1* promoter were slightly lower those of *GFP* alone driven by the *HASP1* promoter, *SF-GFP* levels were still higher than those of the *fcpA* promoter in both the log and stationary phases, suggesting that the presence of the SP sequence somehow affects the steady-state level of GFP mRNA. These results suggest that the *HASP1* promoter is a suitable tool for overexpressing recombinant proteins in *P*. *tricornutum*.

### Relative fluorescence and GFP protein levels in the cell lysates and culture supernatants

**Figure 4:**
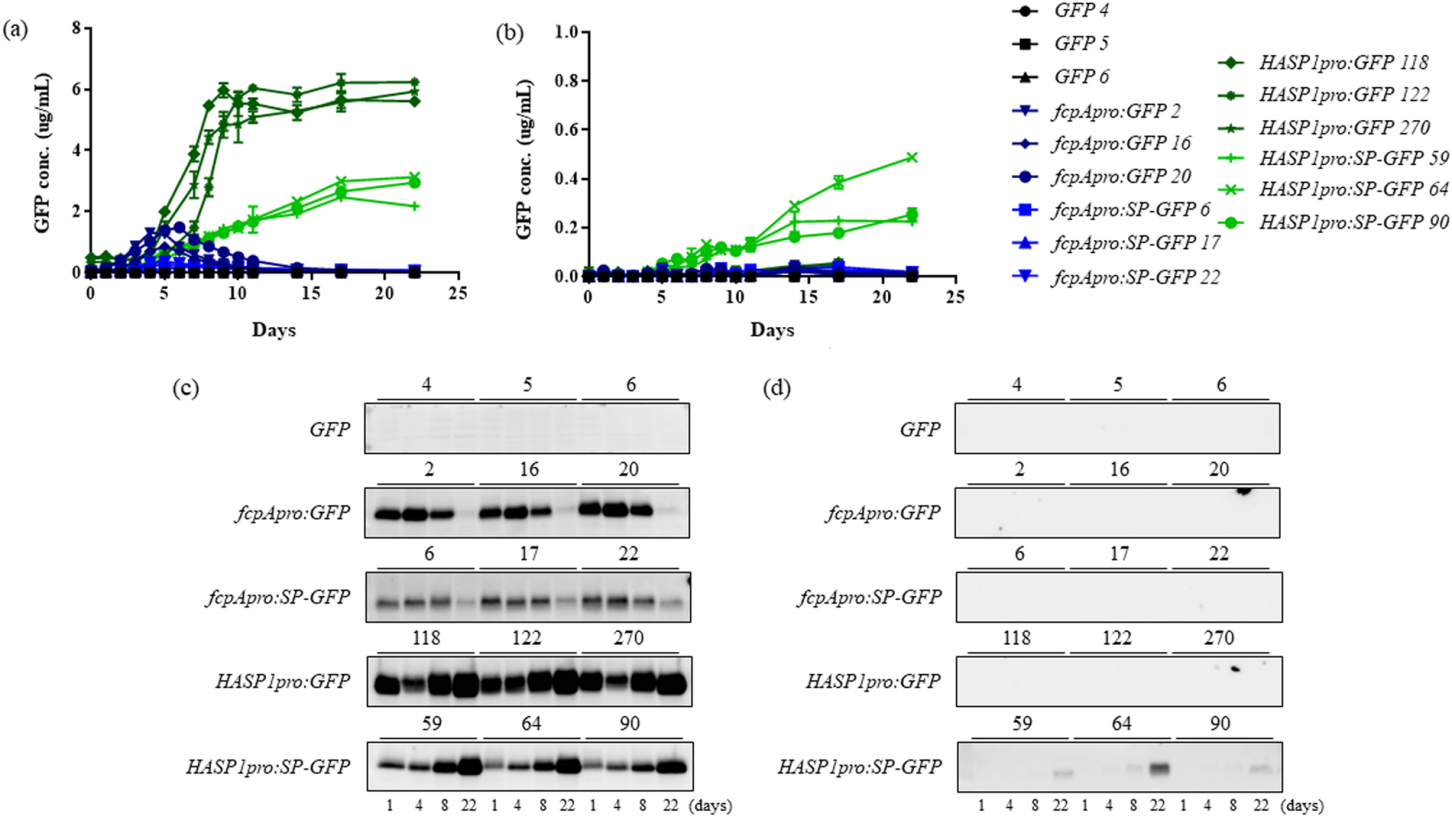
Relative protein expression levels of GFP in the cell lysates and culture supernatants. Levels of GFP fluorescence in (**a**) cell lysates and (**b**) culture supernatants were measured by a fluorometer. The protein levels of GFP in (**c**) cell lysates and (**d**) culture supernatants were determined by immunoblotting. Full-length Western blots are presented in Supplementary Fig. S4.

To assess promoter activities and GFP secretion at the protein level, protein samples were prepared from both the *P*. *tricornutum* cells and culture supernatants. Subsequently, GFP fluorescence was measured using a fluorometer and GFP protein levels were examined by western blotting (Fig. 4).

The fluorescent signal of intracellular GFP in the *fcpApro:GFP* transgenic line gradually increased from the lag to the log phase (day 4) but decreased from the log to the stationary phase (day 8). The intracellular GFP concentration calculated from the fluorescent signal reached a maximal level of 1 µg/mL at the log phase (Fig. 4a). This result supports previous studies that showed that the *fcpA* promoter is maximally active in the log phase and less active in the stationary phase^19–21^. In contrast, the fluorescent signal of intracellular GFP in the *HASP1pro:GFP* transgenic line rapidly increased from the log to the early stationary phase (day 8) which was maintained at a high level throughout the stationary phase until day 22 (Fig. 4a). The intracellular GFP concentration calculated from the fluorescent signal reached a level of 6 µg/mL at the stationary phase. Compared with the *fcpA* promoter, GFP protein levels driven by the *HASP1* promoter were slightly lower in the log phase, but rapidly increased by 6-fold during the early stationary phase, reaching an approximate 300-fold increase by the late stationary phase (Fig. 4a). In contrast, we could not detect GFP fluorescence in the culture supernatants of the transgenic lines expressing *HASP1pro:GFP* or *fcpApro:GFP* due to their lack of a putative signal peptide (Fig. 4b).

To investigate the role of the putative signal peptide of HASP1 in GFP secretion, we measured the fluorescent signals of intracellular and secreted GFP from the same culture of *P*. *tricornutum* over time. The fluorescent signal from intracellular GFP in the *HASP1pro:SP-GFP* transgenic line was similar to that of the *fcpApro:SP-GFP* transgenic line during the log phase but was 3-fold higher during the stationary phase (Fig. 4a). Secreted GFP levels in the *fcpApro:SP-GFP* transgenic line increased slightly to 0.01 µg/mL at the stationary phase (Fig. 4b). In contrast, secreted GFP levels in the *HASP1pro:SP-GFP* transgenic line steadily increased from the log to the stationary phases; extracellular GFP concentrations reached 0.01, 0.1, and 0.3 µg/mL at the log, early stationary, and late stationary phases, respectively. These values correspond to a 3, 8, and 19- fold increase compared with that of the *fcpApro:SP-GFP* transgenic line (Fig. 4b). Moreover, secreted GFP levels under the control of the *HASP1* promoter were comparable to those secreted with the gametolysin signal sequence reported previously in *C*. *reinhardtii*^25^.

To confirm the GFP fluorescence results obtained by the fluorometer, we also performed western blot analysis of cell lysates and culture supernatants over the same time course. With the exception of day 1, protein levels of both intracellular and secreted GFP in the cell lysates and culture supernatants were consistent with the fluorescent signals seen for intracellular and secreted GFP. On day 1, the protein levels of both intracellular GFP and SP-GFP were abnormally high. It should be noted that GFP fluorescence measurements were made with protein samples prepared from the same culture volumes (200 µL), whereas for western blotting, the protein levels of GFP and SP-GFP were determined using the same amount of total soluble protein (10 µg). Intracellular GFP was detected at high levels in all transgenic lines expressing *fcpApro:GFP*, *fcpApro:SP-GFP*, *HASP1pro:GFP*, and *HASP1pro:SP-GFP* except for the promoter-less *GFP* control (Fig. 4c; uncropped Western blot images used to generate Fig. 4c are shown in Supplementary Fig. S4). Protein levels of intracellular GFP driven by the *fcpA* promoter were the highest at the log phase, whereas those by the *HASP1* promoter were the highest at the late stationary phase. In contrast to the intracellular GFP levels, extracellular GFP secreted from the *P*. *tricornutum* cells could only be detected in the *HASP1pro:SP-GFP* transgenic line (Fig. 4d; uncropped Western blot images used to generate Fig. 4d are shown in Supplementary Fig. S4). This result is inconsistent with the GFP fluorescence measurements, likely due to differences in detection limits between a fluorometer and western blotting.

Overall, these data demonstrate that the *HASP1* promoter is much stronger than the *fcpA* promoter, especially during the stationary phase, and that the signal peptide derived from the HASP1 protein is sufficient to facilitate efficient GFP secretion. There have been several studies that tested endogenous and exogenous promoter ability in driving the expression of heterologous genes in *P*. *tricornutum*^7,15,19,41,44–46^. Compared to previously reported promoters, our data provide several lines of evidence that both the promoter and signal peptide of the *HASP1* gene can be powerful tools for the efficient expression and secretion of heterologous recombinant proteins throughout all growth phases of a *P*. *tricornutum* culture.

### Subcellular localisation of GFP with or without the signal peptide of the HASP1 protein

**Figure 5:**
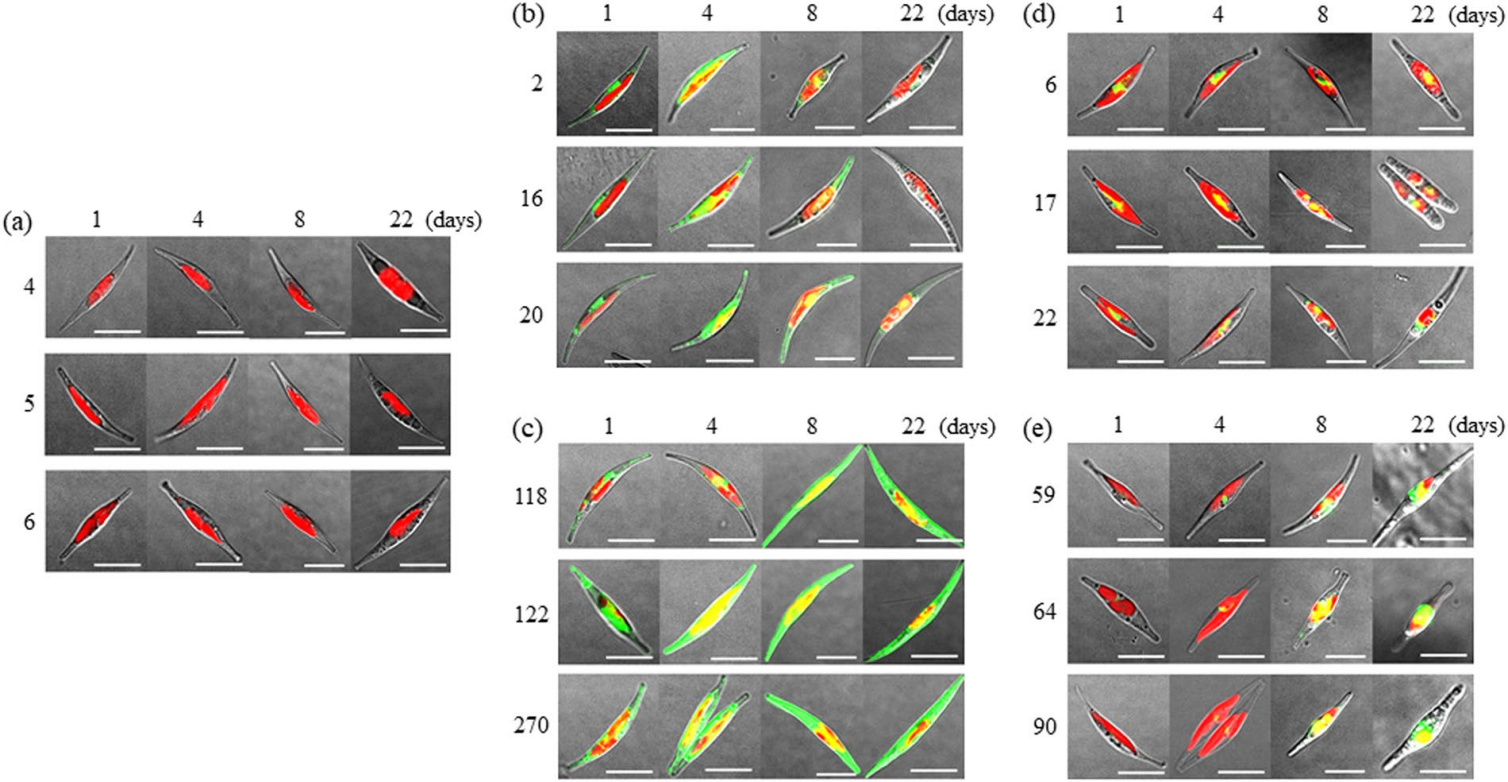
Effect of the HASP1 signal peptide on subcellular localisation of GFP. GFP fluorescence and chlorophyll autofluorescence in transgenic lines expressing the (**a**) promoter-less *GFP*, (**b**) *fcpApro:GFP*, (**c**) *HASP1pro:GFP*, (**d**) *fcpApro:SP-GFP*, and (**e**) *HASP1pro:SP-GFP* were visualised by confocal laser scanning microscopy. The numbers on the left side of the images indicate three independent transgenic lines for each construct. Scale bars = 10 µm.

To observe the subcellular localisation of GFP with or without the HASP1 signal peptide, GFP fluorescence (green) and chlorophyll autofluorescence (red) in all transgenic lines were visualised by confocal laser scanning microscopy. The transgenic line carrying the promoter-less *GFP* was analysed in parallel as a control (Fig. 5a). GFP fluorescence in the *fcpApro:GFP* transgenic line was diffusely localised throughout the cytoplasm at both the log and early stationary phase but suddenly decreased at the late stationary phase (Fig. 5b). In contrast, GFP fluorescence in the *HASP1pro:GFP* transgenic line strongly increased throughout the entire cytoplasm from the lag to the early stationary phase and was maintained at high levels during the stationary phase (Fig. 5c). Interestingly, the signal peptide of HASP1 protein caused considerable changes in the subcellular localisation of GFP during all growth phases. GFP fluorescence in the *fcpApro:SP-GFP* lines during all growth phases was weakly localised near the chloroplast compartment, such as in the chloroplast endoplasmic reticulum^41,47,48^ and a blob-like structure found between the two plastid lobes^47,49,50^ (Fig. 5d). Similarly, GFP fluorescence in the *HASP1pro:SP-GFP* lines was weakly localised near the chloroplast compartment in both the lag and log phase. However, GFP fluorescence in the *HASP1pro:SP-GFP* lines rapidly increased from the log to the early stationary phase (day 8), and was maintained at a high level throughout the stationary phase until day 22 (Fig. 5e). This finding suggests that the HASP1 signal peptide leads to GFP entry into the secretory pathway. Thus far, the pathways utilized for protein secretion in *P*. *tricornutum* remain mostly unknown. Therefore, elucidating the pathways by which proteins are secreted in *P*. *tricornutum* would be an interesting research direction.

## Conclusions

The HASP1 protein is the most abundant protein secreted into the culture medium of *P*. *tricornutum*. Compared with the *fcpA* promoter, the *HASP1* promoter strongly drives GFP expression during all growth phases, especially during the stationary phase. The HASP1 signal peptide also efficiently facilitates GFP secretion into the culture medium. The endogenous *HASP1* promoter as a novel strong promoter can therefore be an alternative to the commonly used *fcpA* promoter. Overall, the combination of the *HASP1* gene promoter and signal peptide demonstrated great potential for improving the production of secreted recombinant proteins by *P*. *tricornutum*.

## Materials and Methods

### Cell culture and cell density measurement

The *P*. *tricornutum* Bohlin UTEX 646 strain was obtained from the UTEX Culture Collection of Algae (The University of Texas at Austin, TX, USA). The strain was grown in 200 mL of f/2 medium^51^. Fifty percent artificial seawater, prepared with Royal Nature Advanced Pro Formula Salt (Royal Nature, Nesher, Israel) and f/2 medium, was autoclaved before addition into the final medium. The vitamin and metal solutions were added after autoclaving. A mixture of antibiotics containing 50 µg/mL ampicillin, 10 µg/mL kanamycin, and 50 µg/mL streptomycin was added to the liquid medium and agar plates to suppress bacterial contamination. Cultures were incubated at 20 °C with shaking at 200 rpm, constant aeration, and continuous lighting from a cool white fluorescent lamp (1600 lux). Cell density measurements at each sampling point were made by measuring the optical density of 200 µL cell culture at 530, 670, and 730 nm (Multi-Detection Microplate Reader Synergy HT; Biotek, Winooski, VT, USA).

### Preparation of secreted proteins from the *P*. *tricornutum* culture in the stationary phase

To profile the proteins secreted by *P*. *tricornutum*, cultures were grown to a density of 1 × 10^7^ cells/mL as described above, and the culture was centrifuged at 3500 rpm for 15 min at 4 °C to pellet the cells. Supernatants were filtered through a 0.2-μm vacuum filter system (Corning Inc., Corning, NY, USA) and concentrated using centrifugal filter devices (Vivaspin 20 and 500; Satorius, Goettingen, Germany) with 5 and 10 kDa cutoff membranes according to manufacturer’s instructions. Protein concentrations were determined by the Bradford assay (Bio-Rad Laboratories, Hercules, CA, USA). Samples were prepared from three independent experiments.

### SDS-PAGE and in-gel digestion

Protein samples (30 μg) were resolved by electrophoresis on 12% SDS-PAGE gels and visualised by Coomassie blue staining. Abundant proteins were excised from the gels and subjected to in-gel digestion^52^.

### LC−MS/MS analysis and protein identification through a database search

LC-MS/MS analysis and protein identification were performed as previously described^19^.

### Construction of plasmid vectors

The pPha-T1 expression vector was used as a backbone for all constructs^30^. The *fcpA* promoter fragment harbouring NdeI/EcoRI restriction sites^30^ and the potential promoter fragment of *HASP1* (Fig. 2) were amplified by PCR from the pPha-T1 vector and *P*. *tricornutum* genomic DNA, respectively. The GFP gene was amplified from a pEGFP-C2 vector with three different restriction enzyme combinations (NheI/BamHI, EcoRI/BamHI, and NdeI/BamHI), which were later used to construct the five different expression vectors. Next, the sequence encoding the putative signal peptide of the HASP1 protein (Fig. 2) was cloned into the constructs using a long primer harbouring EcoRI/NheI restriction sites, the Kozak sequence, the putative signal peptide sequence, and the 5′ end of *GFP*. The promoter-less pPha-T1 vector, harbouring only *GFP*, was used as a negative control. All ligation reactions were performed using a DNA ligation kit (6023; Takara Bio, Kusatsu, Japan). All constructs were verified by sequencing. All primers used in vector construction are listed in Supplementary Table S1.

### Transformation of *P*. *tricornutum* by particle bombardment

The activation of 0.6-μm microcarriers/nanogold particles (9204298; Bio-Rad Laboratories) and DNA coating of microcarriers were performed according to manufacturer’s instructions for the Biolistic^®^ PDS-1000/He Particle Delivery System (165–2257; Bio-Rad Laboratories). *P*. *tricornutum* cells cultivated in 2 L of f/2 medium for 7 days at 20 °C under continuous white fluorescent light (40 μmol·m^−2^·s^−1^) were centrifuged at 3500 rpm for 5 min at 15 °C and then plated on the central third of the f/2 agar plates, which also contained mixed antibiotics. Transformation of the *P*. *tricornutum* cells was conducted as previously described^19^.

### PCR analysis of putative transformants

Harvested *P*. *tricornutum* cells from 20 μL cell culture were lysed in 10 μL of 5 mM Tris-HCl (pH 7.5) buffer containing 0.8% Tween-20^53^. The lysates were centrifuged at 3500 rpm for 5 min at 20 °C and the supernatants were discarded. The pellet was used for PCR analysis of genomic DNA with a Maxime PCR Premix (i-StarTaq; 25167; iNtRON Biotechnology, Seongnam, South Korea). Primers used for PCR analysis are listed in Supplementary Table S1.

### Total RNA preparation and real-time RT-PCR analysis

Five millilitres of cell culture grown for 4 or 8 days was centrifuged at 3500 rpm for 15 min at 4 °C. The pellet was washed twice with 1 mL ice-cold Dulbecco’s phosphate buffered saline (DPBS; Corning™ 21-031-CVR; Thermo Fisher Scientific, Waltham, MA, USA) via centrifugation at 6000 rpm for 5 min at 4 °C. After the last centrifugation step, the pellet was suspended in 1 mL ice-cold DPBS and ground in liquid nitrogen. Total RNA was extracted according to manufacturer’s instructions for the RNAiso Plus Total RNA Extraction Reagent (9109; Takara Bio). First-strand cDNA was then synthesised using a QuantiTect^®^ Reverse Transcription Kit (205311; Qiagen, Hilden, Germany) according to manufacturer’s instructions. Real-time RT-PCR analysis was performed as described in the LightCycler^®^ 480 SYBR^®^ Green I Master (04707516001; Roche Diagnostics, Risch-Rotkreuz, Switzerland) protocol. Primers used for real-time RT-PCR analysis are listed in Supplementary Table S1.

### GFP fluorescence measurement

Protein samples were prepared separately from equal volumes (200 µL) of *P*. *tricornutum* cell culture and culture supernatants for all time points. Fluorescence measurements were performed as previously described^19^. The autofluorescence value of the promoter-less GFP construct was subtracted from the GFP fluorescence value obtained with the *fcpA* and *HASP1* promoters. A GFP standard curve was generated using a recombinant GFP protein (ab119740; Abcam, Cambridge, UK). Measurements were performed with three biological replicates.

### Western blot analysis

Cell lysis was performed as described previously^19^. *P*. *tricornutum* culture supernatant was concentrated as described above. Total soluble protein concentrations were determined using the bicinchoninic acid (BCA) protein assay kit (23225; Thermo Fisher Scientific) according to manufacturer’s instructions. The same amount of total soluble protein (10 μg) in each sample was separated by electrophoresis on 12% homemade Tris-glycine SDS-polyacrylamide gels and then transferred to polyvinylidene difluoride (PVDF) membranes. The membranes were incubated with an anti-GFP goat polyclonal antibody (ab6673; Abcam) and an anti-goat IgG-horseradish peroxidase conjugated bovine polyclonal antibody (sc-2378; Santa Cruz Biotechnology, Dallas, TX, USA). Immunoblot signals were detected using SuperSignal^®^ West Femto Maximum Sensitivity Substrate (34095; Thermo Fisher Scientific).

### Subcellular localisation of GFP

Live cell images were captured using a Leica TCS-SP5 confocal laser scanning microscope (Leica, Wetzlar, Germany). GFP was excited with a 488 nm laser and its fluorescence emission was detected between 509 and 560 nm. Chlorophyll autofluorescence was observed using excitation at 488 nm and emission at 700–750 nm^54^.

### Statistical analysis

Data are expressed as the mean ± SD. Statistical analysis was performed using one-way analysis of variance (ANOVA) followed by Sidak’s multiple comparisons test (GraphPad Prism v7.04). *p* values < 0.05 were considered statistically significant.

## Supplementary information

Supplementary Information

## Data Availability

The datasets generated during and/or analysed during the current study are available from the corresponding author on reasonable request.
