# Supplementary material for: Identification and characterisation of the novel endogenous promoter HASP1 and its signal peptide from Phaeodactylum tricornutum
Source: Sci Rep. 2019 Jul 9;9:9941. doi: 10.1038/s41598-019-45786-9 (PMC6617621; doi:10.1038/s41598-019-45786-9)
Supplement: Supplementary file 1 — Supplementary Information [file 41598_2019_45786_MOESM1_ESM.pdf]

Identification and characterisation of the novel endogenous promoter *HASP1* and its signal peptide from *Phaeodactylum tricornutum*

Erdenedolgor Erdene-Ochir[1,2], Bok-Kyu Shin[3], Byori Kwon[3], Choonkyun Jung[4,*], and Cheol-Ho Pan[1,2,*]

Supplementary Figure S1

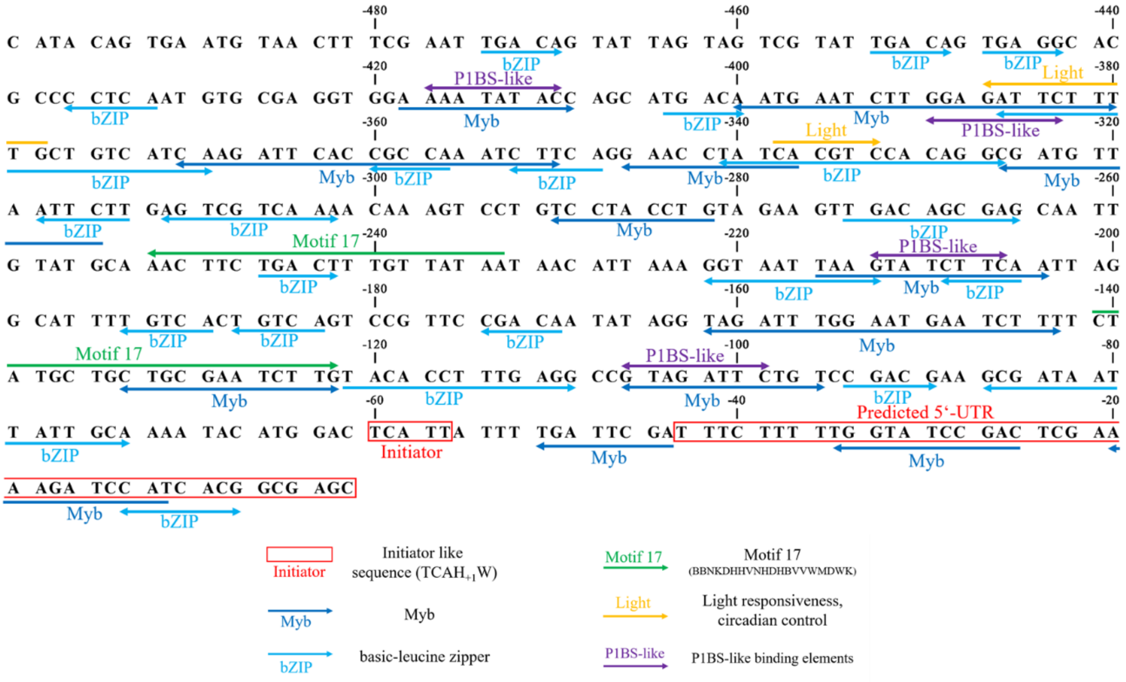

Supplementary Figure S1. *In silico* analysis of potential *cis*-acting regulatory elements in the *HASP1* potential promoter. The 499-bp upstream sequence of the *HASP1* gene was analysed by PlantPAN2.0 and PlantCARE. Motif 17, nitrogen deficiency-responsive element; P1BS-like binding element, PHR1 (phosphate starvation response 1)-binding *cis*-acting element.

Supplementary Figure S2

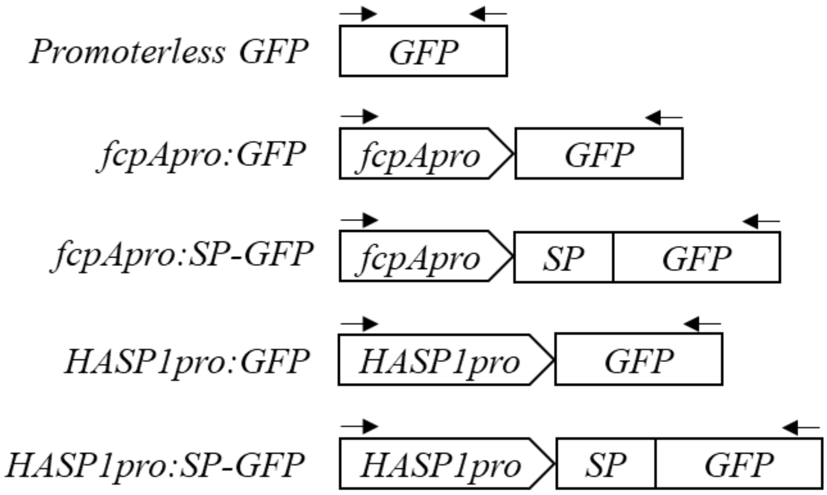

Supplementary Figure S2. Vector constructs used for the transformation of *P. tricornutum*. Arrows indicate the primers used for PCR analysis shown in Fig. 2c. SP, signal peptide.

Supplementary Figure S3

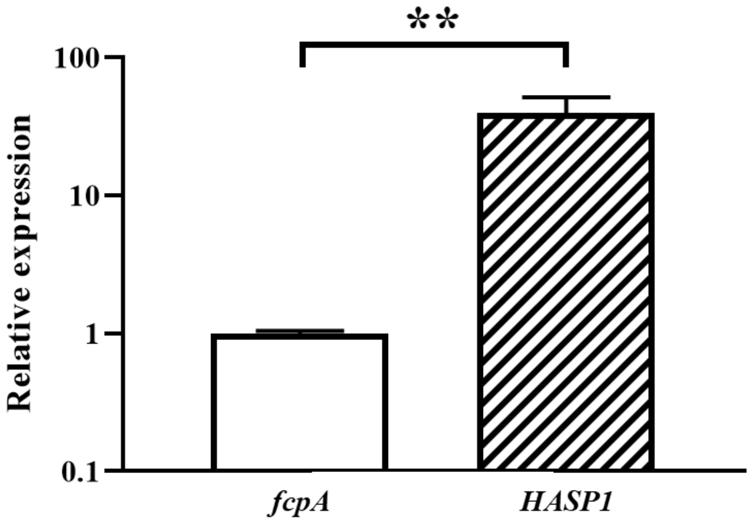

Supplementary Figure S3. Relative levels of endogenous *fcpA* and *HASP1* mRNA in 8-day grown wild-type *P. tricornutum* cells. Transcript levels were normalised to *TBP* (TATA-box binding protein) expression. Data are expressed as the mean ± SD of three replicates. The asterisk indicates statistically significant difference. ** $p < 0.01$ (Unpaired *t*-test).

Supplementary Figure S4

Full-length agarose gels for Fig. 2C

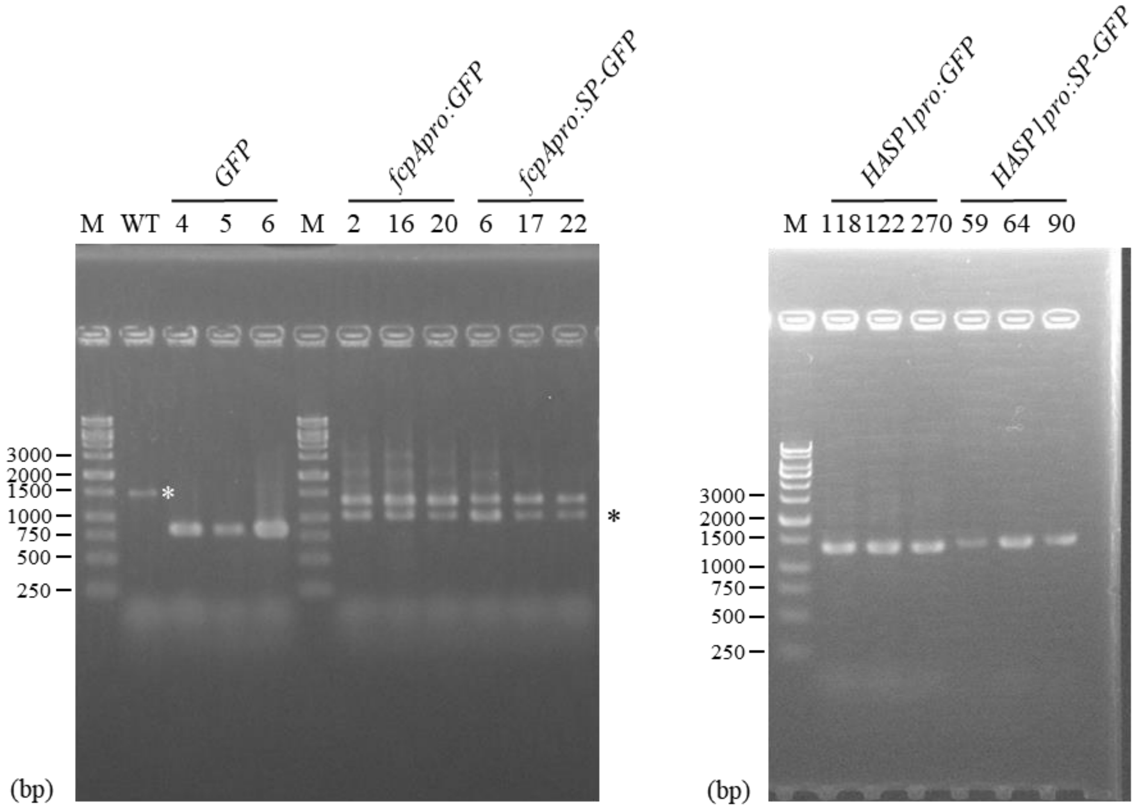

Supplementary Figure S4. Full-length gels and blots shown in the manuscript are provided without cropping. Asterisks indicate the nonspecific PCR products.

Supplementary Figure S4 (continued)

Full-length Western blots for Fig. 4c

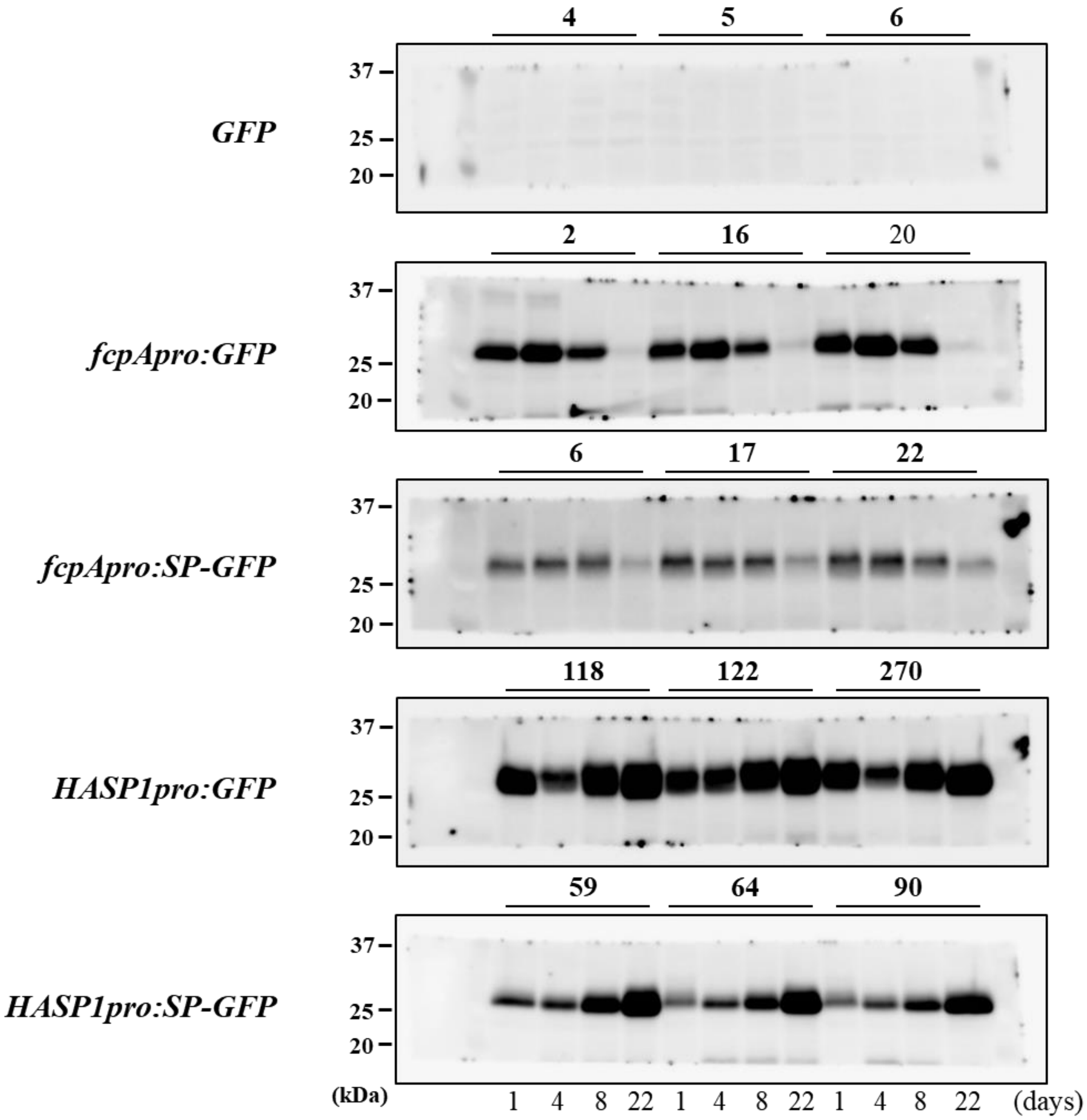

Supplementary Figure S4. After protein transfer, the membranes were cut and incubated with anti-GFP antibody. All blot images were taken at the same exposure time.

Supplementary Figure S4 (continued)

Full-length Western blots for Fig. 4d

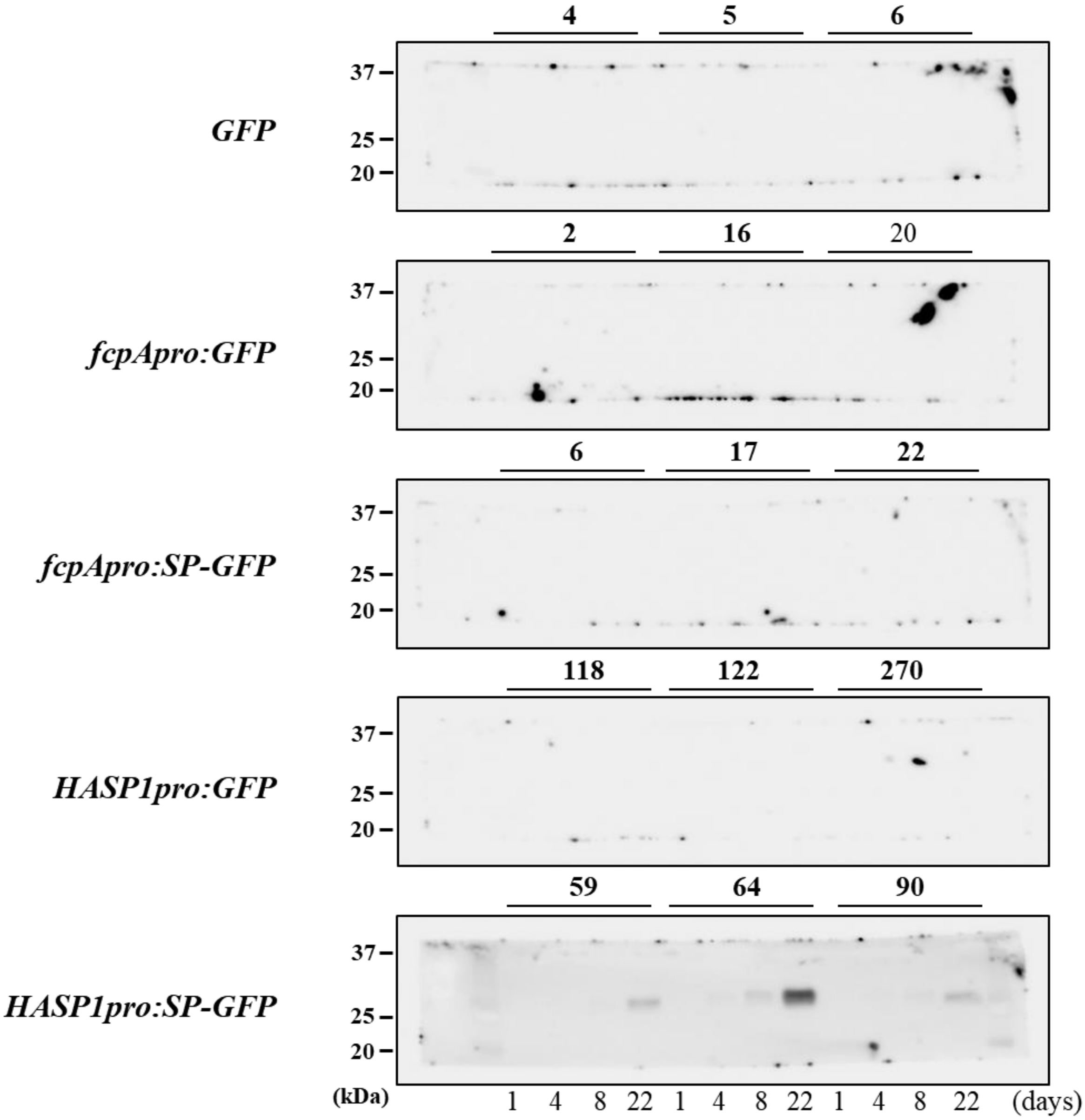

Supplementary Figure S4. After protein transfer, the membranes were cut and incubated with anti-GFP antibody. All blot images were taken at the same exposure time.

Supplementary Table S1

Name	Sequence (5' to 3')	Restriction sites	Description	Reference
F-*fcpA*	CATATGGGGCTGCAGGACGCAATGG	NdeI	pPha-T1-*fcpA* PCR and Genomic DNA PCR	7
R-*fcpA*	GAATTCTCGAAACGGCAGACAAATTTGTG	EcoRI	pPha-T1-*fcpA* PCR	7
F-*HASP1*	CATATGCATACAGTGAATGTAACTTTCG	NdeI	pPha-T1-*HASP1* PCR and Genomic DNA PCR	In this study
R-*HASP1*	GAATTCGCTCGCCGTGATGGATCTTTTC	EcoRI	pPha-T1-*HASP1* PCR	In this study
F-Kozak-SP-GFP	GAATTCACCATGAATCTTCGTTGTATCCTTCCGTTTCTCCTCGCAAGCTTCTCGGCTGGGGCTAGCATGGTGAGCAAGGGCGAGGAG	EcoRI-NheI	Long primer for SP amplification PCR	In this study
F-*gfp*	GCTAGC/GAATTC/CATATGATGGTGAGCAAGGGCGAGGAG	NheI/ EcoRI/ NdeI	pPha-T1-*gfp* PCR	In this study
R-*gfp*	GGATCCTTACTTGTACAGCTCGTCCATGC	BamHI	pPha-T1-*gfp* PCR	In this study
R-pPha-T1-Multi-B	ACTCCCAACTGTTCGTGCACCATG		Genomic DNA PCR	30
F-qRT-GFP	AGAACGGCATCAAGGTGAAC		qRT-PCR	In this study
R-qRT-GFP	TGCTCAGGTAGTGGTTGTCG		qRT-PCR	In this study
F-qRT-TBP	ACCGGAGTCAAGAGCACACAC		qRT-PCR	11
R-qRT-TBP	CGGAATGCGCGTATACCAGT		qRT-PCR	11
F-qRT-fcpA	GTAGCCACCAACATGGCTTT		qRT-PCR	In this study
R-qRT-fcpA	CGAACTTCTCCTGGTTACCG		qRT-PCR	In this study
F-qRT-HASP1	CAATCAGTTCATCGCCATTG		qRT-PCR	In this study
R-qRT-HASP1	CGACCACATTGTTGGATCAG		qRT-PCR	In this study

Supplementary Table S1. Primers used in this study.
